# A Novel Variant of the *CHD2* Gene Associated With Developmental Delay and Myoclonic Epilepsy

**DOI:** 10.3389/fgene.2022.761178

**Published:** 2022-02-11

**Authors:** Lina Zhu, Fujun Peng, Zengwen Deng, Zhichun Feng, Xiuwei Ma

**Affiliations:** ^1^ Faculty of Pediatrics, Chinese PLA General Hospital, BaYi Children’s Hospital, The Seventh Medical Center of PLA General Hospital, Beijing, China; ^2^ School of Basic Medical Sciences, Weifang Medical University, Weifang, China; ^3^ Hospital of Jvlu County, Xingtai, China

**Keywords:** whole-exome sequencing, *CHD2* variants, developmental delay, myoclonic epilepsy, epileptic encephalopathy

## Abstract

Pathogenic variants in *CHD2* have been reported to have a wide range of phenotypic variability in neurodevelopmental disorders, such as early-onset epileptic encephalopathy, developmental delay, and behavior problems. So far, there is no clear correlation between genotypes and phenotypes. This study reports a Chinese patient with a novel heterozygous *CHD2* mutation (c.4318C>T, pArg1440*). Her main clinical manifestations include developmental delay, myoclonic epilepsy, and hypothyroidism. Then, we reviewed a total of 144 individuals carrying *CHD2* variants with epileptic encephalopathy. In terms of clinical manifestations, these patients are usually described with variable epilepsy phenotypes, including idiopathic photosensitive occipital epilepsy, Dravet syndrome, Jeavons syndrome, Lennox–Gastaut syndrome, juvenile myoclonic epilepsy, and non-specific epileptic encephalopathy. Among them, myoclonic seizures and generalized tonic-clonic seizures are the main seizure types in all patients hosting *CHD2* single-nucleotide or indel variants (non-CNVs). At the molecular level, there are 102 types of *CHD2* non-CNVs in 126 patients, almost one mutational type corresponding to one person, and there is no difference in the incidence ratio of each position. Furthermore, we summarized that a small proportion of patients inherited *CHD2* variants, and not all patients with *CHD2* variants had seizures. Importantly, the phenotypes, especially seizures control and fever sensitivity, and genotypes had a relative association. These results enriched the database of CHD2-relative neurodevelopmental disorders and provided a theoretical foundation for researching the relationship between genotypes and phenotypes.

## Introduction

Chromodomain helicase DNA-binding protein 2 (CHD2, MIM: 615369) is a member of the CHD family, which is only known to cause the brain-restricted phenotypes when disrupted in humans. It is mapped to chromosome 15q26.1 and is considered an ATP-dependent chromatin remodeling that regulates the transcription expression of many genes (Lamar and Carvill, 2018; Carvill and Mefford, 2015). Some studies have demonstrated that pathogenic variants of the *CHD2* gene are associated with childhood-onset developmental and epileptic encephalopathy (DEE), which is a severe form of neurodevelopmental disorder with a wide range of phenotypic variability, including autism spectrum disorder (ASD), intellectual disability (ID), developmental delay, microcephalus, behavioral anomalies, facial dysmorphisms, and several types of epilepsy (Carvill and Mefford, 2015; Thomas et al., 2015; Verhoeven et al., 2016; Chen et al., 2020). Recently, Chen et al. presented the largest single case series of patients with CHD2-related epilepsy and then comprehensively reviewed 53 published cases in the literature through seizure onset age, seizure types, developmental outcome, electroencephalogram (EEG), brain magnetic resonance imaging (MRI), and diagnoses, among others, which improved the understanding on the relationship between genotype and phenotype (Chen et al., 2020). In 2021, De Maria et al. reported 18 new patients and reviewed 84 previously reported patients, getting the results that the median age of seizures onset in 92% of patients was 2.5 years, and there was no clear association between genotypes and phenotypes (De Maria et al., 2021).

This study reported a novel variant in the *CHD2* gene in a Chinese girl with developmental delay and myoclonic epilepsy through whole-exome sequencing (WES). We also systematically reviewed the published literature, provided a thorough overview of clinical, neuroimaging, physical, and genetic findings in CHD2-related epilepsy patients, and built the possible relationship between genotypes and phenotypes.

## Materials and Methods

### Case Collection

A total of 59 papers, including 144 cases, were considered candidates (Table 1 and Supplementary Table S1). These cases were collected from studies and databases and reported to associate with the *CHD2* mutations. The main process is as follows: 1) the keywords “CHD2” and “neurodevelopmental disorders” or “epileptic encephalopathy” were used to search the PubMed database for relevant papers; 2) the full text of each eligible publication was downloaded and read carefully. The papers and review articles on these cases were saved; 3) using the “CHD2” gene as the keyword, relevant papers in the HGMD database were acquired, downloaded, screened, and reserved, and these papers were associated with CHD2-related epileptic encephalopathy; 4) the collected review articles were used to confirm the conclusions from the obtained papers and complement the lacking literature; 5) patient information, including clinical information, neuroimaging, physical, and genetic findings, was extracted from these papers, such as gender, inheritance, and diagnostic findings; and 6) all results were checked by more than two researchers, and the opposite consequences were verified after discussion.

**TABLE 1:** The seizure types of patients with *CHD2* mutations.

	Single-nucleotide or indel variants (*n* = 126)	Copy number variants (*n* = 18)
Gender	F (43), M (61), NA (22)	F (11), M (7)
Age	0∼6 years (26), 6∼12 years (27), >12 years (37), NA (36)	0∼6 years (3), 6∼12 years (4), >12 years (8), NA (3)
Inheritance	*De novo* (111), mother (2), father (3), NA (10)	*de novo* (18), mother (0), father (0), NA (0)
Age of seizure onset	≤1 year (16), 1∼6 years (70), 6∼12 years (5), >12 years (2), NA (33)	≤1 year (2), 1∼6 years (11), 6∼12 years (3), >12 years (1), NA (1)
Development before seizure onset	Delay (47), normal (22), NA (57)	Delay (12), normal (0), NA (6)
Seizure control	Yes (18), no (36), NA (72)	Yes (6), no (9), NA (3)
Introduce factors		
Photosensitivity	Yes (45), no (37), NA (44)	Yes (5), no (1), NA (12)
Fever sensitivity	Yes (14), no (37), NA (75)	Yes (0), no (4), NA (14)
Cognition/development outcome		
ASD	Yes (34), no (48), NA (44)	Yes (7), no (5), NA (6)
ADHD	Yes (12), no (48), NA (66)	Yes (1), no (8), NA (9)
ID	Yes (76), no (8), NA (42)	Yes (15), no (3), NA (0)
Behavior problem	Yes (47), no (2), NA (77)	Yes (10), no (0), NA (8)
Other abnormal findings^#^	Yes (78), no (8), NA (40)	Yes (16), no (0), NA (2)
Physical development		
Height	Normal (17), short stature (6), tall stature (1), NA (102)	Normal (4), short stature (6), tall stature (0), NA (8)
Weight	Normal (11), underweight (5), obesity (2), NA (108)	Normal (6), underweight (1), obesity (2), NA (9)
Head circumference	Normal (15), microcephaly (9), NA (102)	Normal (2), microcephaly (3), NA (13)
EEG	Normal (2), abnormal (80), NA (44)	Normal (0), abnormal (9), NA (9)
MRI	Normal (64), abnormal (8), NA (54)	Normal (9), abnormal (5), NA (4)
Facial dysmorphisms	yes (11), no (7), NA (108)	Yes (12), no (0), NA (6)

Abbreviations: d, day; m, month; y, year; ADHD, attention deficit hyperactivity disorder; ASD, autism spectrum disorder; EEG, electroencephalography; F, female; ID, intellectual disability; M, male; MRI, magnetic resonance imaging; NA, not applicable; #, other abnormal findings mainly contained delay in motor and language development, learning disability, illusions or hearing odd sounds, defective social communications, poor balance, short-term memory problems, *etc*.

### Whole-Exome Sequencing

Genomic DNA was extracted from the peripheral blood of all the family members using DNA Isolation Kit (Blood DNA Kit V2, CW2553) according to standard procedure. Whole-exome capture and sequencing were performed using SureSelect Human All Exon V6 (60 Mb) kit (Agilent, Santa Clara, USA, and sequenced on the Illumina Nova series platform (Illumina, San Diego, USA), generating 150 bp paired-end pairs. The raw reads of sequencing underwent the process of trimming, depolluting, and filtering to get only the high-quality reads. Only those that passed these filtrations could be used for the downstream analyses. High-quality paired-end reads were aligned to the human reference genome sequence from the UCSC database (build 37.1 version hg19, http://genome.ucsc.edu/) using the Burrows–Wheeler Alignment tool. We estimated quality scores and made the consensus SNP and insertions and deletions (indels) calling using GATK [1]. All the called variants were annotated with ANNOVAR software to give the variant position, variant type, allele frequency, conservation prediction, and so forth, which would help locate mutations relative to diseases. All variants were filtered using the 1000 Genomes, ExAC, ChES, and gnomAD databases, as well as a minor allele frequency (MAF) ≤ 1%. Then, a series of analyses were used with OMIM database, HGMD database, and phenotypes-genotypes association analysis. Finally, the candidate variants were judged according to the ACMG standard and validated by Sanger sequencing. All samples were obtained with written informed consent from patients.

### Statistical Analyses

All the statistical analyses were performed using SPSS 22.0. Pearson χ2 test was used to assess the significance of differences between groups. In the study of the number of patients with various seizure types (Table 2 and Supplementary Table S2), the average value was introduced, which was equal to the number of all the patients except for unclassified generalized seizures (GS), unclassified seizures, and not applicable (NA) divided by the number of seizure types containing at least one patient. Similarly, the average concept was introduced in the study of the number of patients with various epilepsy types/syndrome (Figure 3 and Supplementary Table S3). In the study of the incidence rate of variants in different domains of *CHD2* (Figure 4A and Supplementary Table S4), the incidence rate was calculated by dividing the mutational numbers in one domain divided by mRNA sequence lengths of this domain, and the average value was equal to the number of all the variants divided by the length of mRNA sequence in *CHD2* gene. In the studies of seizures control, photosensitivity, and fever sensitivity (Figures 4B,C and Supplementary Table S5), the difference values were presented among different domains of CHD2. A two-sided *p*-value < 0.05 was considered statistically significant and was adjusted by the Bonferroni correction.

**TABLE 2:** Clinical characteristics of persons with a *CHD2* mutation in published cases.

	Single-nucleotide or indel variants			Copy number variants		
**Seizure types**	**First**	**Further**	**All**	**First**	**Further**	**All**
aAS	3	**11** ^b^	**14** ^b^	0	0	0
AbS	5	**13** ^b^	**18** ^b^	5	0	5
aMAS	**0** ^a^	0	**0** ^a^	1	0	1
AtS	4	**15** ^b^	**19** ^b^	0	0	0
CSE	2	3	5	0	0	0
DA	**0** ^a^	1	1	0	0	0
EMA	8	4	**12** ^b^	1	1	2
EMs	**0** ^a^	1	1	0	1	1
ES	2	0	2	0	0	0
FCS	**0** ^a^	0	**0** ^a^	0	1	1
FoS	3	**15** ^b^	**18** ^b^	1	0	1
FoSID	1	0	1	1	0	1
FS	14	5	**19** ^b^	3	0	3
GCS	**0** ^a^	0	**0** ^a^	0	1	1
GTCS	**17**	**30** ^b^	**47** ^b^	2	5*	6*
HD	**0** ^a^	2	2	0	1	1
MA	1	6	7	0	0	0
MAS	3	8	10	0	0	0
MCS	**0** ^a^	1	1	0	0	0
MS	**24** ^b^	**30** ^b^	**52** ^b^	0	4	4
NCSE	**0** ^a^	10	10	0	1	1
NS	**0** ^a^	1	1	0	0	0
SE	**0** ^a^	8	8	0	0	0
TCS	5	7	12	0	0	0
TS	1	**11** ^b^	12	0	2	2
Unclassified GS	4	0	4	3	1	3
Unclassified seizures	33	0	28	1	0	1
NA	1	67	0	0	10	0

Abbreviations: aAS, atypical absence seizures; AbS, absence seizures; aMAS, atypical myoclonic-absence seizures; AtS, atonic seizures; CNVs, copy number variants; CSE, convulsive status epilepticus; DA, drop attack; EMA, eyelid myoclonia with absence; EMs, eyelid myoclonias; ES, epileptic spasm; FCS, febrile clonic seizures; FoS, focal seizures; FoSID, focal seizure with impairment of awareness; FS, febrile seizures; GCS, generalized clonic seizure; GS, generalized seizures; GTCS, generalized tonic-clonic seizures; HD, head drops; MA, myoclonic absence seizures; MAS, myoclonic-atonic seizures; MCS, myotonic-clonic seizures; MS, myoclonic seizures; NA, not applicable; NCSE, non-convulsive status epilepticus; NS, non-epileptic seizures; SE, status epilepticus; TCS, tonic-clonic seizures; TS, tonic seizures; *, the difference did not exist using the Bonferroni correction.

a tendence and *p*-value <0.05;

b resistance and *p*-value <0.05.

### Clinical Report

**FIGURE 1:**
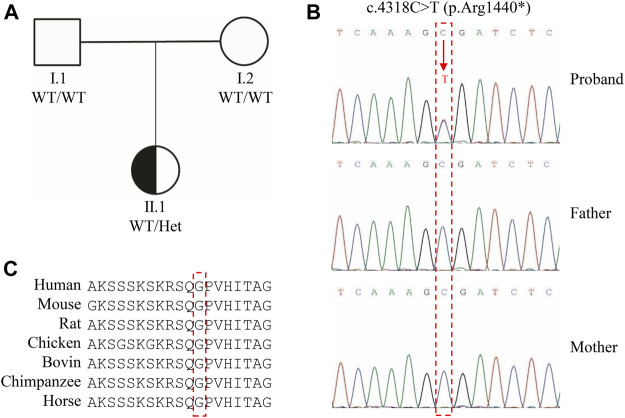
Genetic characterization of the proband. **(A)** Family pedigree. **(B)** Analysis of CHD2 c.4318C>T (NM_001271.4, p. Arg1440*) in a non-related Chinese family. **(C)** Homology alignment of the protein encoded by CHD2.

**FIGURE 2:**
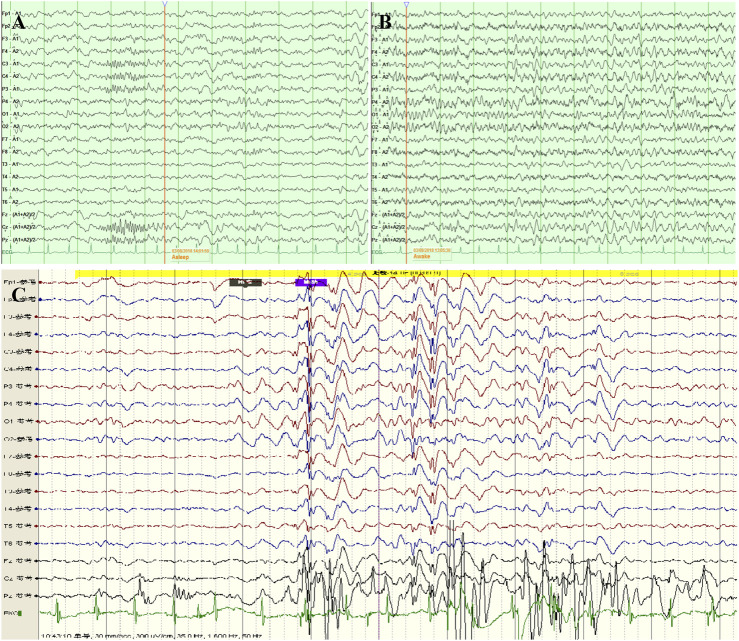
Video electroencephalogram (VEEG) of the proband with development disorders. **(A)** During the sleeping period, a small amount of medium amplitude sharp waves and sharp slow waves were emitted asynchronously in bilateral frontal, central, parietal, and right occipital regions, and sporadic in the right anterior temporal region. **(B)** During the awake period, there had 7-8 Hz low amplitude, a, and Θ mixed waves in the bilateral occipital area. **(C)** When she had myoclonic seizures, the results showed a generalized polyspike wave.

The proband was a 3-year and 2-month old girl, who was a full-term baby born by a healthy and unrelated Chinese couple through cesarean section, weighing 3,900 g, without intrauterine distress, suffocation, and neonatal jaundice (Figure 1A). Three days after birth, a thyroid function test suggested hypothyroidism, and the child took thyroxine tablets regularly to maintain normal thyroid function. She started to roll over at 6 months and sit at 8 months. At 17 months, she could not walk alone, and her Development Screen Test (DST) presented DQ < 70 and MI < 70. The DQ scores of Gesell Developmental Assessment (GDA) are listed below: gross movement is 71, fine movement is 76, adaptive skills score is 71, language is 53, and social score is 71. Brain magnetic resonance imaging (MRI) revealed no severe abnormalities. Then, the patient was required to do a series of rehabilitation training, which resulted in her ability to walk alone at 1.5 years, and run and navigate stairs at 2.5 years. However, she still could not jump or execute instructions and presented poor balance. The development delay was shown in her intelligence and language; for example, she spoke a single word at 2 years and said two or three words at 3 years. She liked to run around and open her mouth and did not like to play with other children. At the age of 2 years and 8 months, she started to experience seizures, and there was no obvious cause such as photosensitivity and fever sensitivity. The seizures were manifested by a rapid shaking of the entire body, which relieved in 1-2 s and occurred several times per day. After remission, the mental reaction was as usual. There was no special family history. The video electroencephalogram (VEEG) showed high-amplitude spike waves, and slow spike waves were burst throughout the brain, especially in the frontal, central, and middle posterior temporal regions (Figures 2A,B). When she had seizures, VEEG showed a generalized polyspike wave (Figure 2C). Other auxiliary examinations results were normal and mainly contained electrocardiogram, blood routine, urine routine, stool routine, biochemistry, myocardial enzymes, thyroid function, blood ammonia, lactic acid, blood tandem mass spectrometry analysis of amino acids and acylcarnitine, and urine organic acid analysis. Oral anti-epileptic treatment with sodium valproate was gradually increased to 30 mg/kg·d, and seizure was controlled.

The girl came to our hospital because of mental retardation with seizures when she was 3 years and 2 months old. She presented normal facial features, active behavior, poor execution, and lacking eye contact with people. The physical examination results revealed a total length of 96 cm, weight of 15 kg, and head circumference of 49 cm. The cardiopulmonary and abdominal examinations showed no abnormality. Dystonia of limbs did not exist, sputum culture reflexes were drawn symmetrically, and pathological signs were negative. The second DQ scores were a gross movement of 60, fine movement of 69, adaptive skills of 66, language of 50, and social score of 67. Brainstem auditory evoked response (BAER) showed a delay in the peripheral conduction of the binaural auditory pathway. Visual evoked potential (VEP) showed that the incubation period of bilateral P100 was prolonged. Integrating the above information, the girl was diagnosed with developmental delay and myoclonic epilepsy.

### Genetic Analysis

Whole-exome sequencing was performed on the proband and her parents. Overall, more than 8 Gb of the sequence generated per patient covered all the exome and splice site regions of genes. The proband revealed a *de novo* heterozygous variant in *CHD2* (c. 4318C>T, p. Arg1440*). This nonsense variant was localized in the 30th exon out of 39 total exons in the *CHD2* gene (NM_001271.4) and was predicted to cause premature termination of translation. In addition, the mutation does not exist in the 1000 Genomes, ExAC, ChES, and gnomAD databases (Macdonald et al., 2014; Lek et al., 2016), and to our knowledge, it has not been reported in affected individuals in the published literature. This variant was also validated by Sanger sequencing. Meanwhile, the *CHD2* genes of her parents were normal (Figures 1A,B). No other significant variants were found in the other genes. Multiple sequence alignment analysis revealed that the p. Arg1440* substitution occurred on an amino acid residue which was evolutionarily highly conserved among different species (Figure 1C).

## Discussion

The CHD protein family (containing CHD1–CHD9) is mainly involved in the ATP-dependent chromatin remodelers that contribute to the reorganization of chromatin structure and deposition of histone variants necessary for regulating gene transcription expression. Among the nine CHD family members, the *CHD2* pathogenic variants only lead to a brain-restricted phenotype when disrupted in humans, which indicates a unique role for this gene in neurodevelopment (Lamar and Carvill, 2018).

*CHD2* mutation-related epilepsy was first published in 2009 (Veredice et al., 2009). This paper described a 30-month-old girl with refractory myoclonic epilepsy associated with mental retardation, growth delay, peculiar facial appearance, minor physical anomalies, and photosensitivity. In the patient, a 15q26.1–15q26.2 deletion including *CHD2* was detected by CGH-array. To date, there are 144 reported individuals encompassing 126 single-nucleotide or indel variants (non-CNVs) and 18 copy number variants (CNVs) with CHD2-related epileptic encephalopathy (Table 1 and Supplementary Table S1). From Table 1 and Supplementary Table S1, almost all patients were diagnosed at over 1 year or even over 6 years of age. Up to now, most *CHD2* variants have been reported *de novo* in patients with epilepsy rather than in an inherited mode. Nevertheless, in 2015, the first patients diagnosed with eyelid myoclonia with absence (EMA) inherited it from an unaffected mother, who carried the *CHD2* variant c.653C>T (p. Pro218Leu) (Galizia et al., 2015). In 2018, the patient who inherited a pathogenic *CHD2* variant c.628G>T (p. Glu218*) from the affected mother was published (Petersen et al., 2018). The distressing thing is that compared with her daughter’s refractory epilepsy, her seizures have always been well controlled (Petersen et al., 2018). In 2020, Chen et al. reported a sporadic case inherited from an unaffected father with c.5153+2T>C variant of *CHD2* gene and dizygotic twins inherited from the affected father with c.5232G>A (p. Met1744Ile) (Chen et al., 2020). To our surprise, the five mutations in patients did not occur again in other cases. In *CHD2* CNVs patients, the inherited patients were not reported. These findings indicate that *CHD2* variants, with the exception of *CHD2* CNVs, can be inherited (Table 1).

Previously, *CHD2* variants were generally considered to be childhood-onset epileptic encephalopathy. However, more and more individuals with *CHD2* mutants were published, and the ages of seizure onset were different from infancy to childhood (Table 1 and Supplementary Table S1). We had also found that different patients had different development in pre-seizure onset. For example, Carvill et al. reported that two out of six patients with epileptic encephalopathies caused by *CHD2* mutations had normal development and another four were abnormal (Carvill et al., 2013). Interestingly, all the patients with *CHD2* CNVs could lead to developmental delays in pre-seizure onsets, such as motor and speech developmental delays (Table 1 and Supplementary Table S1). In addition, the inducing factors, including photosensitivity and fever sensitivity, EEG results, and facial dysmorphisms, were also various among *CHD2* variants’ patients. Galizia et al. performed photosensitivity tests on zebrafish larvae with *CHD2* gene knockout and found that the *CHD2* gene knockout significantly enhanced the photosensitivity of congenital zebrafish larvae. Their results had confirmed that this gene was related to photosensitive epilepsy (Galizia et al., 2015). Except for the twin patients mentioned above, most patients had abnormal EEG results (Chen et al., 2020). We also found that the reported patients had behavior problems such as aggressive behavior. In other aspects of patients, the findings showed diversity, such as ASD, height, and MRI (Table 1 and Supplementary Table S1).

**FIGURE 3:**
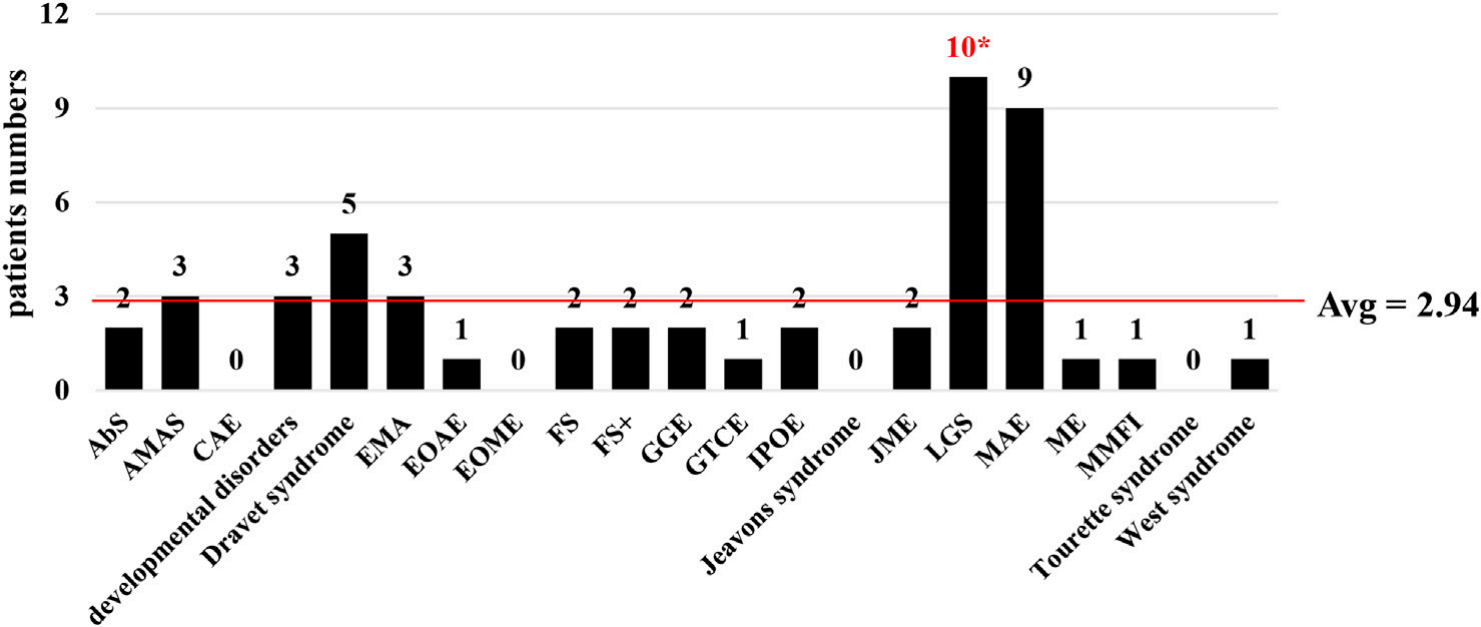
Statistical analysis of all reported CHD2-non-CNVs patients with epilepsy. Distributions of patients according to epilepsy syndrome in the overall cohort, including 126 non-CNVs patients. The epilepsy syndrome did not contain DEE, non-specific GE, non-specific EE, and unclassified epilepsy. The average value = patients‘ numbers of epilepsy syndrome/numbers of epilepsy types. Abbreviations: AbS, absence seizures; CAE, childhood absence epilepsy; DEE, developmental and epileptic encephalopathy; EE, epileptic encephalopathy; EMA, eyelid myoclonia with absence; EOAE, early-onset absence epilepsy; EOME, early-onset myoclonic epilepsy; FS, febrile seizures; FS+, febrile seizure plus; GE, generalized epilepsy; GGE, genetic generalized epilepsy; GTCE, generalized tonic-clonic epilepsy; IPOE, idiopathic photosensitive occipital epilepsy; JME, juvenile myoclonic epilepsy; LGS, Lennox‐Gastaut syndrome; MAE, myoclonic atonic epilepsy; ME, myoclonic epilepsy; MMFI, malignant migrating focal seizures in infancy; non-CNVs, single-nucleotide or indel variants; *, the difference did not exist using the Bonferroni correction.

The patients have variable seizure types, including absence seizures (AbS), atonic seizures, tonic-clonic seizures, and myoclonic absence seizures. Importantly, an individual can have multiple seizure types; even the first seizure and the later seizure could be of different types (Supplementary Table S1). Based on these phenomena, we summarized and analyzed the seizure types of patients with *CHD2* non-CNVs and CNVs (Table 2 and Supplementary Table S2). In the *CHD2* non-CNVs patients, both the myoclonic seizures (MS, 27.3%, 24/88) and generalized tonic-clonic seizures (GTCS, 19.3%, 17/88) were the top two types of the first seizures, which presented the difference than average value (5.87 people/seizure types). Some seizure types, including atypical myoclonic-absence seizures (aMAS, 0%) and febrile clonic seizures (FCS, 0%), did not exist. In contrast, MS (50.8%, 30/59), GTCS (50.8%, 30/59), atonic seizures (AtS, 25.4%, 15/59), focal seizures (FoS, 25.4%, 15/59) and andother three seizure types were the main types of second and later seizures compared to average value (2.95 people/seizure types). In general, MS, GTCS, AtS, febrile seizures (FS), absence seizures (AbS), FoS, atypical absence seizures (aAS), and eyelid myoclonia with absence (EMA) were the main seizure types in *CHD2* non-CNVs (Table 2 and Supplementary Table S2). Interestingly, the main type for *CHD2* CNVs patients could be GTCS, which did not exist using the Bonferroni correction. This phenomenon could be related to the small samples. Furthermore, the patients’ numbers in different epileptic encephalopathy were analyzed using the above methods (Figure 3 and Supplementary Table S3). The results showed that Lennox–Gastaut syndrome (LGS) was usually diagnosed in *CHD2* non-CNVs patients, which presented the difference compared to the average value (2.94 people/epileptic encephalopathy) using the Pearson χ2 test, and the adjusted difference with the Bonferroni correction was disappearance (Supplementary Table S3). The *CHD2* CNVs individuals did not exist in these results (Supplementary Table S3).

**FIGURE 4:**
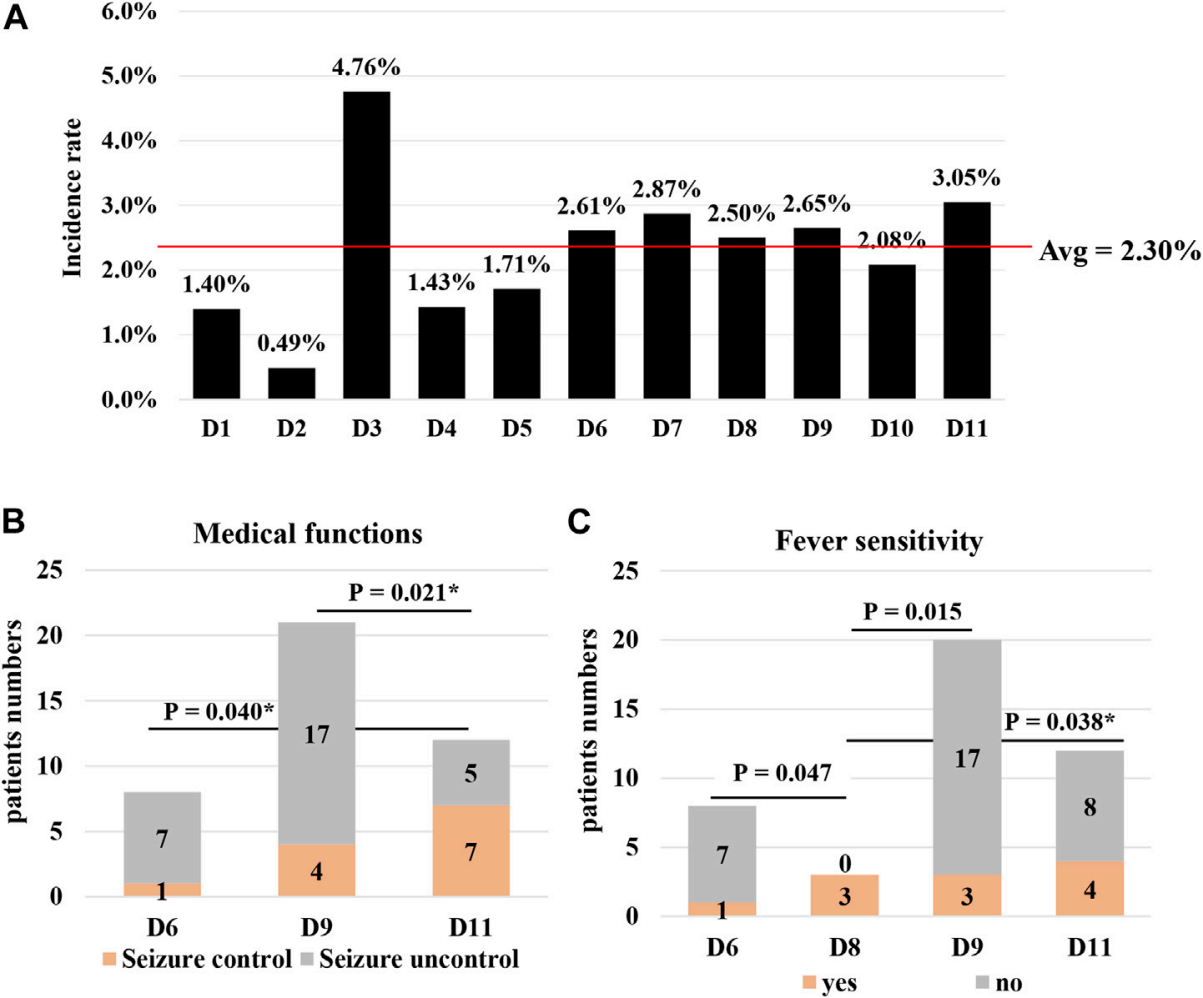
Association analysis between genotypes and phenotypes in *CHD2* non-CNVs patients. **(A)** Distributions of the incidence rate of 126 non-CNVs in different *CHD2* domains. The incidence rate was the mutational numbers in one domain divided by mRNA sequence lengths of this domain and the average value equal to all the variants numbers divided by mRNA sequence lengths of the *CHD2* gene. **(B,C)** The association analysis between *CHD2* genotypes in different domains and phenotypes including seizure control and fever sensitivity in *CHD2* non-CNVs patients. Abbreviations: non-CNVs, single-nucleotide or indel variants; reference protein sequences, NP_001262.3; *, the difference did not exist using Bonferroni correction; D1, 1-262aa; D2, 263-330aa, chromodomain; D3, 331-337aa; D4, 378-447aa, chromodomain; D5, 448-486aa; D6, 487-767aa, SNF2 family; D7, 768-825aa; D8, 826-905aa, helicase conserved C-terminal domain; D9, 906-1458aa; D10, 1459-1554aa, domain of unknown function; D11, 1555-1828aa.

Though a series of studies indicated that there was no clear correlation between genotypes and phenotype in CHD2-related epileptic encephalopathy (Epi et al., 2013; Chen et al., 2020; De Maria et al., 2021), we thought that it was necessary to further research the association between genotypes and phenotypes. We referred to the mRNA sequences NM_001271.4 and protein sequences NP_001262.3 to analyze all the *CHD2* non-CNVs related epilepsy patients (Thomas et al., 2015; Carvill and Mefford, 2015) (Figure 4 and Supplementary Tables S4, S5). The whole protein sequences were divided into 11 fragments (D1–D11), including four conserved domains (Thomas et al., 2015). There were 102 types of *CHD2* non-CNVs in 126 patients, and almost one mutational type corresponded to one patient. Among them, the c.4173dupA (p. Gln1392Thrfs*17) and c.5035C>T (p. Arg1679*) could be the hotspot variants but only occurred five times, respectively (Supplementary Table S4). The *CHD2* variants were distributed in the whole protein sequences and focused on the D9 fragments (906-1458aa, 33.92%, 37/126) (Supplementary Table S4), which led to a serious shortcoming because the longer the domain, the more the number. In order to address this shortcoming, the incidence rate was applied. Though the D11 fragments (1554-1828aa, 3.05%, 25/825) tended to host the *CHD2* variants, the difference value was not shown than the average value (2.30%) (Figure 4A and Supplementary Table S4). Meanwhile, The D3 fragment was too short and was not further analyzed. Interestingly, the incidence rate presented the difference between D1 and D11 (Supplementary Table S4). We also analyzed the relationship between *CHD2* genotypes and seizure control, inducing factors including photosensitivity and fever sensitivity in all the *CHD2* non-CNVs patients, and found a significant correlation between genotypes and phenotypes (Figures 4B,C and Supplementary Table S5). The patients with *CHD2* variants in D11 tended to have better seizure control, and patients with *CHD2* variants in D6 and D9 tended to have worse seizure control. Similarly, there also was a difference in fever sensitivity among D6, D8, D9, and D11. The patients in D8 tended to have more serious fever sensitivity compared to D6, D9, and D11. Though the difference for photosensitivity did not present with *CHD2* variants in D6, D9, and D11, patients with *CHD2* variants in D9 suffered photosensitivity more easily than *CHD2* variants in D6 and D11. These results suggested that there could be associations between genotypes and phenotypes, which were further demonstrated by the dizygotic or monozygotic twins (Pinto et al., 2016; Wang et al., 2017; Chen et al., 2020). Pinto et al. reported that the monozygosity cases had the same seizure onset at 2 years and 6 months old, carried the c.4173dupA (p. Gln1392Thrfs*17) mutations, and presented a set of similar clinical features phenotypes, including autism spectrum disorder, hypotonia, postnatal microcephaly, stereotypic movements, circadian rhythm alterations, and AbS (Pinto et al., 2016). Wang et al. also reported that the monozygotic twins had the same mutation c.5035C>T (p. Arg1679*) and showed similar clinical features (Wang et al., 2017). In another paper, the dizygotic twins were diagnosed with febrile seizure plus (FS+), carried the c.5232G>A (p. Met1744Ile) mutation inherited from the affected father, showed similar clinical descriptions such as fever introduce, MRI normal, EEG normal, first seizure type GTCS, and seizure control except for seizure onset and further seizure type (Chen et al., 2020).

A large number of studies demonstrated the phenotypic heterogeneity of CHD2-associated epilepsy. So far, 144 patients have been reported to have epileptic seizures and associated *CHD2* variants (Table 1 and Supplementary Table S1). However, not all patients with *CHD2* variants have seizures. Up to now, a total of 9 patients with *CHD2* variants were reported to have no epilepsy (Bhakta et al., 2005; Kulkarni et al., 2008; Chenier et al., 2014; Hamdan et al., 2014; Pinto et al., 2014; Chen et al., 2017; Cabrera-Salcedo et al., 2019), For example, Hamdan et al. found that a severe ID girl with motor and speech development delays and *CHD2* variant c.335C>G (p. Ser112*) did not have the history of epilepsy (Hamdan et al., 2014). Interestingly, patients without epilepsy tended to be *CHD2* CNVs patients (Bhakta et al., 2005; Kulkarni et al., 2008; Chenier et al., 2014; Hamdan et al., 2014; Pinto et al., 2014). So far, five patients carrying *CHD2* CNVs but without epilepsy have been reported. For example, Pinto et al. reported a patient with a *de novo* deletion in the *CHD2* gene, with ASD, mild ID, and dysmorphic features including micrognathia and protruding ears, but no seizures (Pinto et al., 2014). However, his ASD-affected brother carried the same deletion of the *CHD2* gene, had similar dysmorphic features and mild ID, and experienced an epilepsy onset at 9 years of age. Kulkarni et al. described a *de novo* translocation t(X; 15) (p22.2; q26.1) dn disrupting *CHD2* in a child with developmental delay, scoliosis, and hirsutism (Kulkarni et al., 2008).

According to the data, seizures in about 1/4 of patients are controlled through single or multiple anti-epileptic drugs (Table 1 and Supplementary Table S1). Interestingly, the ages of seizure onset of patients who were seizures control and carried *CHD2* non-CNVs were from 1 to 12 years, and generalized tonic-clonic seizures (GTCS, 61.1%, 11/18) and myoclonic seizures (MS, 38.9%, 7/18) were the top two seizure types (Supplementary Table S1). However, most patients with CHD2-related neurodevelopmental disorders remain refractory to treatment. Although in some epilepsy syndromes, a ketogenic diet could have vital benefits (Daci et al., 2018; Zhou et al., 2018), a total of four patients with *CHD2* variants were treated with the ketogenic diet, which did not produce significant effects (Thomas et al., 2015; Costain et al., 2019). Therefore, a large sample size and follow-up studies would be helpful to define the treatment and prognosis of CHD2-related epilepsy.

## Conclusion

In this study, a girl with developmental delay and myoclonic epilepsy caused by a new mutation c.4318C>T (pArg1440*) in the *CHD2* gene was studied using WES. The mutation produced a nonsense variant and disrupted the CHD2 protein structure.

Subsequently, all patients with reported neurodevelopmental disorder and variants in the *CHD2* gene were systematically reviewed and analyzed. A total of 144 patients, including 126 non-CNVs and 18 CNVs with *CHD2* pathogenic variants, were analyzed. We found that, except for *CHD2* CNV, a small portion of patients obtained *CHD2* variants by inheritance rather than *de novo*. The ages of seizure onset with *CHD2* mutants varied from infancy to childhood, even adults. The patients with *CHD2* non-CNVs had a certain tendency toward the seizure types, such as MS and GTCS. Meanwhile, not all patients with *CHD2* variants had seizures. Although almost one mutational type corresponded to one person, there may be an association between genotypes and phenotypes, especially for seizure control and fever sensitivity. The above results can provide a theoretical basis for better research on CHD2-related neurodevelopmental disorders.

## Data Availability

The datasets for this article are not publicly available due to concerns regarding participant/patient anonymity. Requests to access the datasets should be directed to the corresponding author.
